# Testicular ACE regulates sperm metabolism and fertilization through the transcription factor PPARγ

**DOI:** 10.1016/j.jbc.2023.105486

**Published:** 2023-11-20

**Authors:** Tomohiro Shibata, Shabir A. Bhat, DuoYao Cao, Suguru Saito, Ellen A. Bernstein, Erika Nishi, Juliet D. Medenilla, Erica T. Wang, Jessica L. Chan, Margareta D. Pisarska, Warren G. Tourtellotte, Jorge F. Giani, Kenneth E. Bernstein, Zakir Khan

**Affiliations:** 1 Department of Pathology and Laboratory Medicine, Cedars-Sinai Medical Center, Los Angeles, California, USA; 2 Department of Biomedical Sciences, Cedars-Sinai Medical Center, Los Angeles, California, USA; 3 Division of Reproductive Endocrinology and Infertility, Department of Obstetrics and Gynecology, Cedars-Sinai Medical Center, Los Angeles, California, USA; 4 Department of Neurology, Cedars-Sinai Medical Center, Los Angeles, California, USA

**Keywords:** testicular ACE (tACE), sperm, metabolism, ATP, fertilization, male fertility, PPARγ

## Abstract

Testis angiotensin-converting enzyme (tACE) plays a critical role in male fertility, but the mechanism is unknown. By using ACE C-domain KO (CKO) mice which lack tACE activity, we found that ATP in CKO sperm was 9.4-fold lower than WT sperm. Similarly, an ACE inhibitor (ACEi) reduced ATP production in mouse sperm by 72%. Metabolic profiling showed that tACE inactivation severely affects oxidative metabolism with decreases in several Krebs cycle intermediates including citric acid, cis-aconitic acid, NAD, α-ketoglutaric acid, succinate, and L-malic acid. We found that sperms lacking tACE activity displayed lower levels of oxidative enzymes (CISY, ODO1, MDHM, QCR2, SDHA, FUMH, CPT2, and ATPA) leading to a decreased mitochondrial respiration rate. The reduced energy production in CKO sperms leads to defects in their physiological functions including motility, acrosine activity, and fertilization in *vitro* and in *vivo*. Male mice treated with ACEi show severe impairment in reproductive capacity when mated with female mice. In contrast, an angiotensin II receptor blocker (ARB) had no effect. CKO sperms express significantly less peroxisome proliferators–activated receptor gamma (PPARγ) transcription factor, and its blockade eliminates the functional differences between CKO and WT sperms, indicating PPARγ might mediate the effects of tACE on sperm metabolism. Finally, in a cohort of human volunteers, in *vitro* treatment with the ramipril or a PPARγ inhibitor reduced ATP production in human sperm and hence its motility and acrosine activity. These findings may have clinical significance since millions of people take ACEi daily, including men who are reproductively active.

Angiotensin-converting enzyme (ACE) is a zinc-dependent dipeptidyl carboxypeptidase. There are two isoforms of ACE: somatic ACE (sACE) and testicular ACE (tACE). These isoforms are transcribed from the same gene through the action of alternative promoters. sACE is composed of two homologous catalytic domains (N domain and C domain), while tACE is approximately half the size of sACE and contains only the C-domain (1). tACE plays a critical role in fertilization in that absence of tACE causes defects in sperm passage through the oviduct and in binding to the zonae pellucidae (1, 2, 3). Several studies demonstrated that tACE affects sperm motility (4, 5, 6), capacitation (7, 8), the acrosome reaction (8), and sperm-oocyte fusion (9). However, the mechanism by which ACE regulates sperm motility and fertilization is not known.

The C-domain of ACE cleaves angiotensin I to angiotensin II (Ang II) which increases blood pressure. Millions of people take ACE inhibitor (ACEi) or Ang II-AT1R blockers (ARBs) daily for treating hypertension, diabetes, and other cardiovascular diseases. Some of these patients are young adults who fall in an active reproductive age group (below 45 years old) (10). Therefore, evaluating the effect of ACEi on sperm functions and fertilization is clinically very important. However, mice lacking angiotensinogen (source for all angiotensin peptides produced by ACE) have normal fertility, indicating that neither Ang II nor any other ang peptide mediate the biological function of tACE in sperm (2).

Sperm motility and fertilization are heavily dependent on energy metabolism. ATP energy is not only required for axonemal dynein (a cytoskeletal motor protein) to drive sperm motility (11, 12), but also essential for sperm capacitation and fertilization (13). Sperm, rich in mitochondria, use oxidative phosphorylation as the major source of ATP production. Not surprisingly, mitochondrial activity (site of oxidative phosphorylation) correlates with sperm motility and capacitation (14). Mitochondrial defects are one of the causes of asthenospermia in men, resulting in low sperm motility and infertility in patients (15). Our group is investigating the metabolic role of both tACE and sACE. We found that C-domain activity of ACE affects ATP production in myeloid cells and thus influences their immune response (16, 17, 18, 19, 20). This led us to investigate the role of tACE in sperm metabolism and whether it is associated with sperm physiological functions. Our study shows that tACE is required for normal energy production in sperm. Specifically, tACE increases mitochondrial ATP production by inducing oxidative phosphorylation, which in turn influences sperm motility and fertilization. The level of peroxisome proliferators–activated receptor gamma (PPARγ) is very low in C-domain KO (CKO) sperm and WT sperm treated with ACEi compared with WT sperm with normal ACE activity. Blockade of PPARγ activity eliminates metabolic and functional differences between CKO and WT sperm, suggesting PPARγ mediates the effect of tACE on sperm. A reduction in the number of pregnant mice was also observed when mice were treated with an ACEi. Furthermore, in human sperm, ACEi treatment reduced ATP production and impaired physiological functions. These studies indicate that tACE regulates sperm functions through energy production and that ACEi treatment may reduce male fertility.

## Results

### tACE is required for normal ATP production in sperm

**Figure 1:**
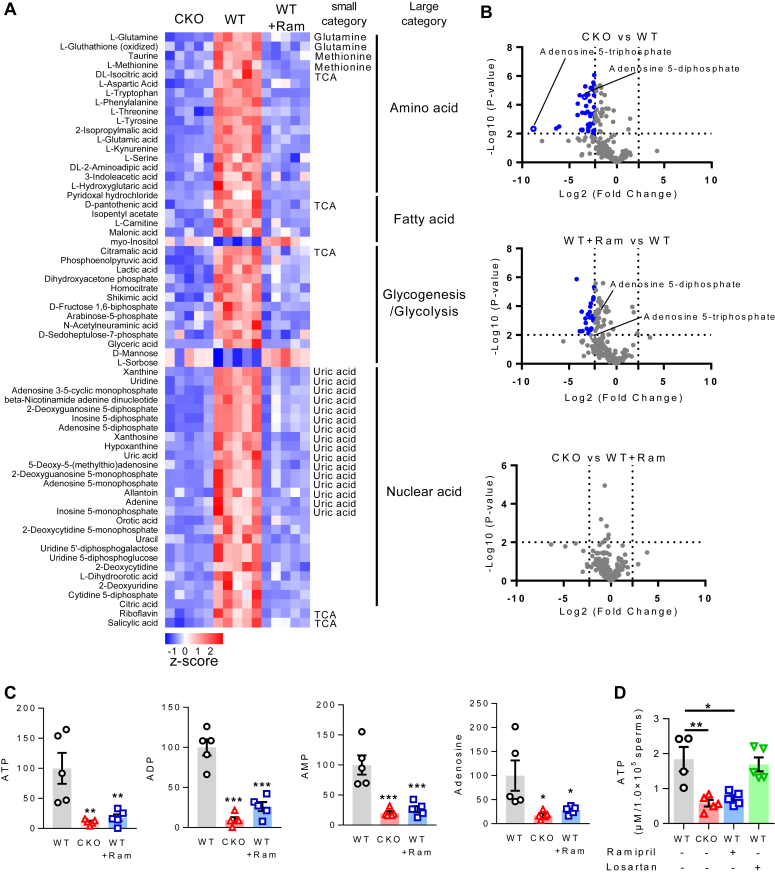
**The effect of tACE on sperm metabolites.***A*, heatmap showing metabolites that are significantly changed (*p*-value < 0.01) among two groups: CKO *versus* WT and WT+Ram *versus* WT. Mean z-scores were created for each protein using GraphPad Prism version 7.04. The mean data for all metabolites are listed in Table S1. WT+Ram groups of mice were treated with 40 mg/l ramipril (Ram) for 1 week before sperm isolation. *B*, volcano plots showing the metabolites that are significantly changed in CKO *versus* WT, WT+Ram *versus* WT and CKO *versus* WT+Ram. The *blue dots* represent downregulated metabolites. The differentially expressed metabolites were sorted with the criteria of *p*-value < 0.01 and Fold Change > 5. *C*, differential cellular levels of ATP, ADP, AMP, and adenosine in WT, CKO, and WT+Ram sperm. *A*–*C*, metabolite array was performed using mass spectrometry (n = 5/group). *D*, biochemical analysis of ATP production in WT and CKO sperm. Indicated groups of mice were treated with either ramipril (40 mg/l) or losartan (600 mg/l) for a week before sperm isolation. *A*–*D*, a one-way ANOVA with Bonferroni’s correction for multiple comparisons was used to analyze group comparisons, and data are presented as means ± SEM. ∗*p* < 0.05, ∗∗*p* < 0.01 and ∗∗∗*p* < 0.001. CKO, C-domain KO; tACE, testis angiotensin-converting enzyme.

To assess the effect of tACE on mouse sperm metabolism, we first determined the levels of intermediate metabolites in CKO and WT sperm by mass spectrometry (20). We found that 64 metabolites were significantly different between CKO and WT sperm (*p* < 0.01). Sixty-one metabolites were low in CKO sperm, while three metabolites (mannose, sorbose, and myo-inositol) were high in CKO sperm than WT (Fig. 1*A*). Similarly, we also found a significant reduction in carbon metabolite production (*e.g.* citric acid, aspartic acid, malic acid, and glyceric acid) in WT sperm when treated with the ACEi ramipril. Importantly, the amount of AMP, ADP, and ATP was decreased in CKO and ramipril treated WT sperm as compared to untreated WT sperm. In CKO sperm, the levels of ATP, ADP, AMP, and adenosine were respectively, 9.4, 10.4, 4.9, and 5.7-fold lower than WT sperm (Fig. 1, *B* and *C*, *p* < 0.05). To verify the mass spectrometry data, the level of cellular ATP was measured by chemical assay in additional samples with or without treatment with ramipril. Again, we found a 3.1-fold reduction of ATP in CKO sperm thanthe WT sperm. Ramipril eliminated the difference between CKO and WT (Fig. 1*D*). Similar experiments were performed using losartan, an AT1 receptor antagonist, to determine the role of the Ang II AT1 receptor. Losartan had no effect on ATP production in CKO and WT sperm (Fig. 1*D*), indicating that Ang II AT1 receptor does not mediate the effect of tACE on sperm ATP production.

### tACE affects mitochondrial proteins regulating energy production

**Figure 2:**
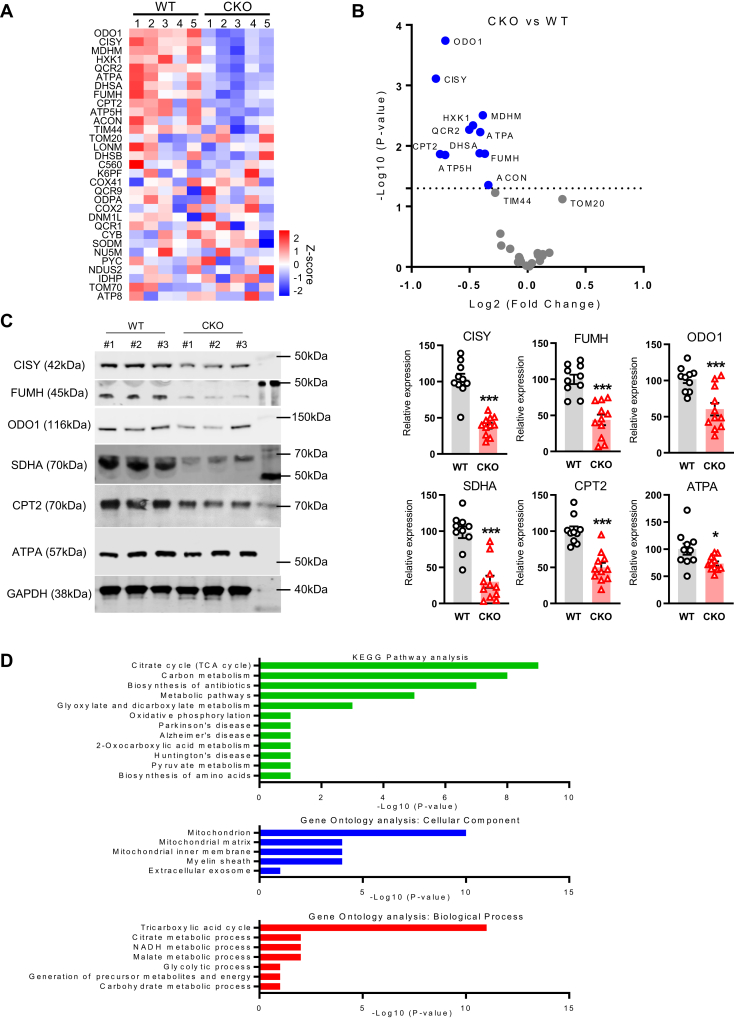
**The effect of tACE on mitochondrial protein in sperm.***A*, heat map showing the level of mitochondrial proteins in WT and CKO sperm measured by the MitoPlex assay. Mean z-scores were created for each protein using GraphPad Prism version 7.04. Data are from the analysis of sperm (5 mice/group). The mean data for all proteins are listed in Table S2. *B*, volcano plot showing differential level of proteins between CKO and WT sperm. The *blue dots* represent downregulated mitochondrial proteins. The significantly different proteins are sorted according to the criteria of *p*-value < 0.05. *C*, measurement of selected mitochondrial proteins by Western blot analysis. Data are presented as means ± SEM (n = 10/group). ∗*p* < 0.05; ∗∗∗*p* <0.001 determined by two-tail student *t* test. *D*, KEGG pathway and GO analyses of mitochondrial proteins using the MitoPlex array. A significant difference between WT and CKO sperm is determined by the *p*-value less than 0.05. CKO, C-domain KO; GO, Gene Ontology; KEGG, Kyoto Encyclopedia of Genes and Genomes; tACE, testis angiotensin-converting enzyme.

Mitochondrial metabolic pathways are the major source of ATP production in animal cells including sperm. Since tACE affects ATP production in sperm, to assess whether tACE affects mitochondrial functions, we measured mitochondrial proteins using a mass spectrometry MitoPlex panel (21). This assay determines the levels of 37 mitochondrial proteins critical to central carbon chain metabolism and overall mitochondrial function. We found that 11 mitochondrial proteins were significantly decreased in CKO sperm as compared to WT sperm, including oxoglutarate dehydrogenase (ODO1), citrate synthase (CISY), malate dehydrogenase (MDHM), hexokinase 1 (HXK1), succinate dehydrogenase complex subunit A (SDHA), fumarate hydratase (FUMH), carnitine palmitoyl transferase II (CPT2), cytochrome b-c1 complex subunit 2 (QCR2) and ATP synthase subunit alpha (ATPA) (Figs. 2, *A* and *B* and S1, *p* < 0.05). MitoPlex data were further validated by Western blot analysis, which identified CKO sperm to be significantly depleted of mitochondrial proteins as compared to WT sperm (Figs. 2*C* and S2). Importantly, most of these proteins are associated with oxidative metabolism.

**Figure 3:**
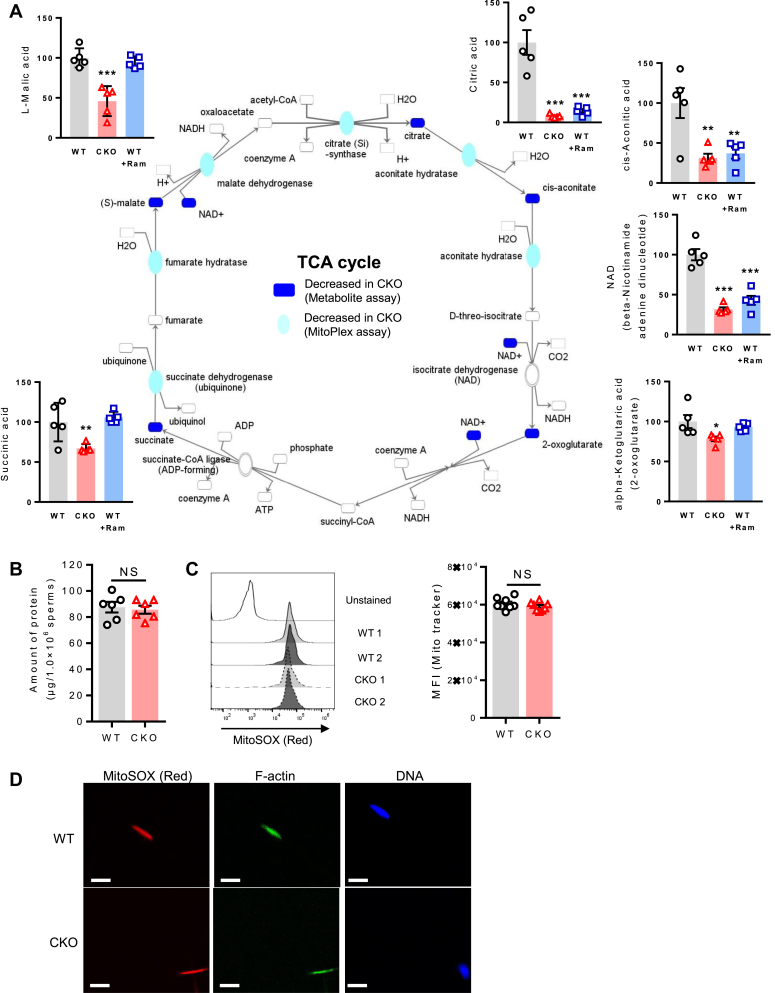
**The measurement of the Krebs cycle in CKO and WT sperm.***A*, measurement of intermediate metabolites and mitochondrial proteins of the Krebs cycle in sperm using metabolite and MitoPlex arrays. The Krebs cycle was analyzed using ingenuity pathway analysis. *Blue shading* represents decreased levels of metabolites (*p* = 0.05) or mitochondrial proteins (*p* = 0.01). Data for this analysis have been taken from the mass spectrometry analysis shown in Figures 1*A* and 2*A*. *B*, the total amount of protein per million sperm measured by the BCA assay. *C* and *D* the mitochondrial content/size of sperm is measured by staining them with MitoSOX dye (Mitotracker *Red*) and DAPI (*blue*). *C*, stained samples were analyzed by flow cytometry. *Left*: representative histograms. *Right*: graph showing mean fluorescent intensity (MFI). Each *dot* represents data from one mouse. *D*, samples were examined by microscopy (5 μm scale bar). *A*–*D*, two-sided unpaired Student *t* test was used to analyze comparisons, and one-way ANOVA with Bonferroni’s correction for multiple comparisons was used to analyze group comparisons. ∗*p* < 0.05; ∗∗*p* < 0.01; ∗∗∗*p* < 0.001. BCA, bicinchoninic acid; CKO, C-domain KO; DAPI, 4′,6-diamidino-2-phenylindole; NS, no significance.

To determine which exact biological processes can be influenced by tACE activity, we carried out two different analyses, Kyoto Encyclopedia of Genes and Genomes pathway analysis and Gene Ontology analysis using MitoPlex data (Fig. 2*D*). These analyses showed that the Krebs cycle was significantly affected in CKO sperm (Fig. 2*D*). As part of metabolite profiling by mass spectrometry, we determined the levels of intermediate metabolites of the Krebs cycle, which showed that CKO sperm produced significantly lower levels of Krebs cycle intermediates, including citric acid (12.6-fold), *cis*-aconitic acid (3.1-fold), NAD (3.1-fold), α-ketoglutaric acid (1.3-fold), succinate (1.5-fold), and L-malic acid (2.2-fold) (Fig. 3*A*), as compared to WT sperm. Thus, these results indicate that the Krebs cycle is one of the main metabolic pathways influenced by tACE activity.

To examine whether differences in ATP, Krebs cycle metabolites, and mitochondrial proteins were due to differences in overall cellular protein content, the amount of protein per cell was determined chemically, we found no difference in protein content between CKO and WT sperm (Fig. 3*B*). We also determined mitochondrial number and morphology (size) by staining mitochondria with MitoSOX red dye and analyzing them by flow cytometry and confocal microscopy. The fluorescent intensity parallels the count and integrity of mitochondria in the sperm midpiece. Again, we found no difference between CKO and WT sperm (Fig. 3, *C* and *D*). This suggests that tACE influences mitochondrial metabolism rather than its biogenesis.

### PPARγ mediates the effect of tACE on sperm mitochondrial metabolism

**Figure 4:**
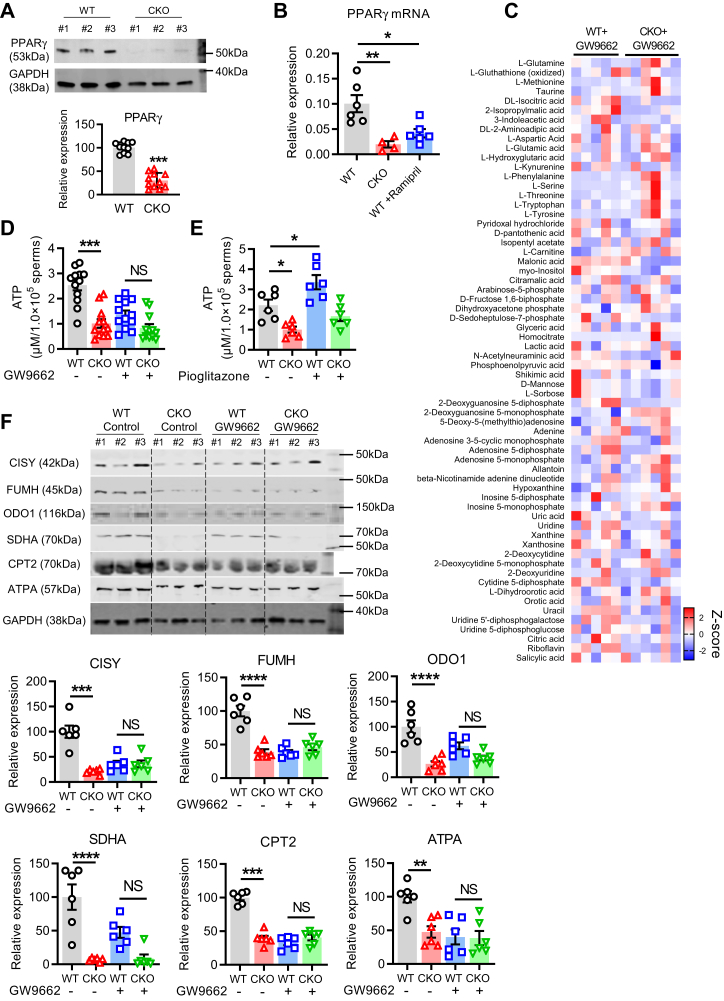
**The role PPARγ in the metabolic effect of tACE in sperm.***A*, Western blot showing PPARγ level in WT and CKO sperm (*left*). Quantitative data (relative levels after β-actin-corrected) (n = 10/group). *B*, the measurement of the PPARγ mRNA level using qRT-PCR in CKO and WT sperm ± WT mice treated with ramipril (40 mg/l) for one week. *C*, heat map illustrating mass spectrometry metabolites array data obtained from WT and CKO sperm after 12 h treatment with 10 μM GW9662. The significantly changed metabolites were sorted according to the criteria of *p*-value < 0.05 and Fold Change >2 among the two groups. The mean data for all metabolites are shown in Table S3. *D*, measurement of ATP levels in sperm after 12 h treatment with or without GW9662 (10 μM; n = 12/group). *E*, measurement of ATP levels in sperm following 12 h treatment with or without Pioglitazone (10 μM; n = 6/group). *F*, Western blot showing the level of mitochondrial proteins in WT and CKO sperm after 12 h treatment with 10 μM GW9662 (n = 6/group). *A*–*F*, data are presented as means ± SEM. Two-sided unpaired Student *t* test was used to analyze comparisons, and one-way ANOVA with Bonferroni’s correction for multiple comparisons was used to analyze group comparisons. ∗*p* < 0.05, ∗∗*p* < 0.01; ∗∗∗*p* < 0.001; ∗∗∗∗*p* < 0.0001. CKO, C-domain KO; NS, no significance; PPARγ, peroxisome proliferators–activated receptor gamma; qRT-PCR, quantitative real time PCR; tACE, testis angiotensin-converting enzyme.

PPAR family proteins are closely associated with mitochondrial function and energy homeostasis (22). Particularly PPARγ has been reported to control energy production and thus is important for sperm physiological functions including motility, the acrosin activity, and survival (23, 24, 25, 26, 27). To examine whether tACE affects the protein level of PPARγ, we performed Western blot analysis of CKO and WT sperm protein lysates and found a significantly reduced level of PPARγ in CKO sperm as compared to WT sperm (Figs. 4*A* and S3). Transcriptional activity is repressed during spermiogenesis; however, the various mRNAs are transcribed for spermiogenesis in advance before terminating of nuclear transcription (28). Therefore, to investigate how tACE increases PPAR levels, we first determined PPARγ transcription (mRNA level). We found that the mRNA level of PPARγ was significantly decreased in CKO sperm as compared to WT sperm (Fig. 4*B*). Then we also assessed the protein stability of PPARγ and the degradation rate by the proteasome using cycloheximide (translation inhibitor) or MG-132 (proteasome inhibitor). However, there was no difference between WT and CKO sperm (Fig. S4).

Next, we examined if PPARγ mediates the effect of tACE on sperm metabolism. First, we measured the level of metabolites in CKO and WT sperm pretreated with PPARγ inhibitor GW9662 for 12 h using mass spectrometry. As shown by heatmap analysis, we found no difference in intermediate metabolites between CKO and WT sperm treated with the PPARγ inhibitor (Fig. 4*C*). In both CKO and WT sperm, ATP levels were decreased after PPARγ inhibition, and there was no difference between CKO and WT groups (Fig. 4*D*). In contrast, PPARγ agonist treatment significantly increased ATP production in WT sperm than the untreated group, while no effect was found on CKO sperm (Fig. 4*E*). Thus, agonist and antagonist had opposite effects on energy production in WT sperm. However, both had no significant effect on CKO sperm due to a minimal PPARγ level.

Next, we determined the mitochondrial proteins in CKO and WT sperm treated with GW9662 using Western blot analysis and found that PPARγ inhibition eliminated the difference between CKO and WT sperm. Specifically, the levels of mitochondrial proteins related to oxidative metabolism including CISY, FUMH, ODO1, SDHA, ATPA, and CPT2 were decreased and similar in both CKO and WT sperm after treatment (Figs. 4*F* and S3). These data suggest that PPARγ plays a central role in the tACE-mediated regulation of mitochondrial metabolism.

### tACE induces ATP production *via* oxidative phosphorylation

**Figure 5:**
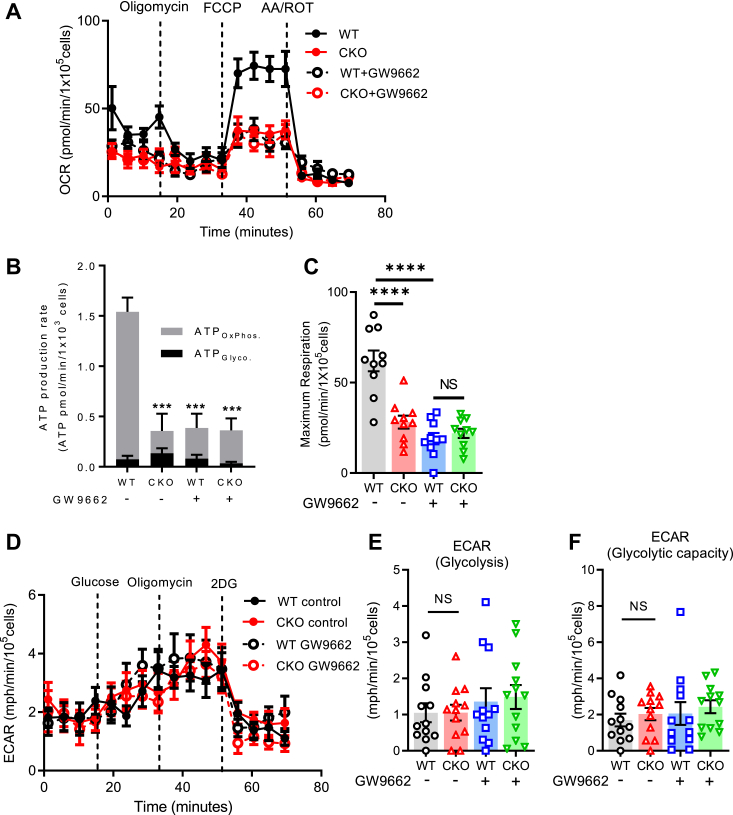
**tACE induces mitochondrial respiration.***A*, oxygen consumption rates (OCR) of WT or CKO sperm measured with an Agilent MitoXpress oxygen consumption assay. The graph shows the trace of OCR in sperm under basal conditions and in response to mitochondrial effectors oligomycin, FCCP, and antimycin A/rotenone. *B*, the rates of ATP production from glycolysis (ATP_Glyco._) and oxidative phosphorylation (ATP_OxPhos._) in WT and CKO sperm. *C*, OCR of maximal respiration (n = 10/group). *D*, extracellular acidification rate (ECAR) of WT or CKO sperm. The data show the trace of ECAR in sperm under basal conditions and in response to glucose, oligomycin, and 2-deoxy-d-glucose (2DG). *E* and *F*, ECAR of glycolysis (*E*) and glycolytic capacity (*F*) (n = 12 per group). *A*–*F*, groups of samples were treated with 10 μM GW9662 for 12 h before analysis as indicated. Data are presented as means ± SEM. An one-way ANOVA with Bonferroni’s correction for multiple comparisons was used to analyze group comparisons. NS, no significance. ∗∗*p* < 0.01 and ∗∗∗*p* < 0.001. CKO, C-domain KO; ECAR, extracellular acidification rate; FCCP, carbonyl cyanide 4-(trifluoromethoxy) phenylhydrazone; tACE, testis angiotensin-converting enzyme.

Because tACE is required for normal production of ATP and Krebs cycle intermediates, we examined whether there was a difference in mitochondrial function between WT and CKO sperm. For this, we measured cellular respiration rate in live cells using the Agilent MitoXpress oxygen consumption assay. After seeding and adhering sperm to a XF96 plate, respiratory parameters were measured as described previously (20, 29, 30). The rates of total ATP production and ATP production by oxidative metabolism (ATP_OxPhos._) in CKO sperm were about 7-fold less than WT sperm (0.22 pmol *versus* 1.47 pmol; *p* < 0.001, n = 10); this difference between the groups was eliminated when the assay was performed in the presence of the PPARγ inhibitor GW9662 (Fig. 5, *A* and *B*). However, there was no difference in ATP production by glycolysis (ATP_Glyco._) between CKO and WT sperm (Fig. 5, *A* and *B*). To confirm if the difference in oxygen consumption between CKO and WT sperm was due to direct mitochondrial changes, we measured maximal oxygen consumption rates (OCRs) with carbonyl cyanide 4-(trifluoromethoxy) phenylhydrazone (FCCP). After blocking ATP synthase with oligomycin, the addition of FCCP measures maximal respiratory rates when mitochondria are uncoupled from ATP synthesis. Indeed, there was a clear difference in maximal oxygen consumption, which averaged 55% lower in CKO sperm than the WT sperm (28.2 ± 10.6 pmol of O_2_/min/1.0 × 10^5^ sperm (CKO) *versus* 64.2 ± 17.1 pmol of O_2_/min/1.0 × 10^5^ sperm (WT), *p* < 0.01) (Fig. 5*C*). Since glycolysis is another major pathway of ATP production in sperm, we further verified whether tACE influences glycolysis by directly measuring extracellular acidification rate (ECAR), which reflects the rate of glycolysis. We found no difference in glycolysis and glycolytic capacity between CKO and WT sperm (Fig. 5, *D*–*F*). Also, we did not observe a significant effect of PPARγ inhibition on glycolysis in these groups (Fig. 5, *D*–*F*). These results indicate that tACE specifically induces ATP production by mitochondrial oxidative phosphorylation, but not by glycolysis, and PPARγ mediates these effects.

### tACE regulates sperm physiological function and fertilization

**Figure 6:**
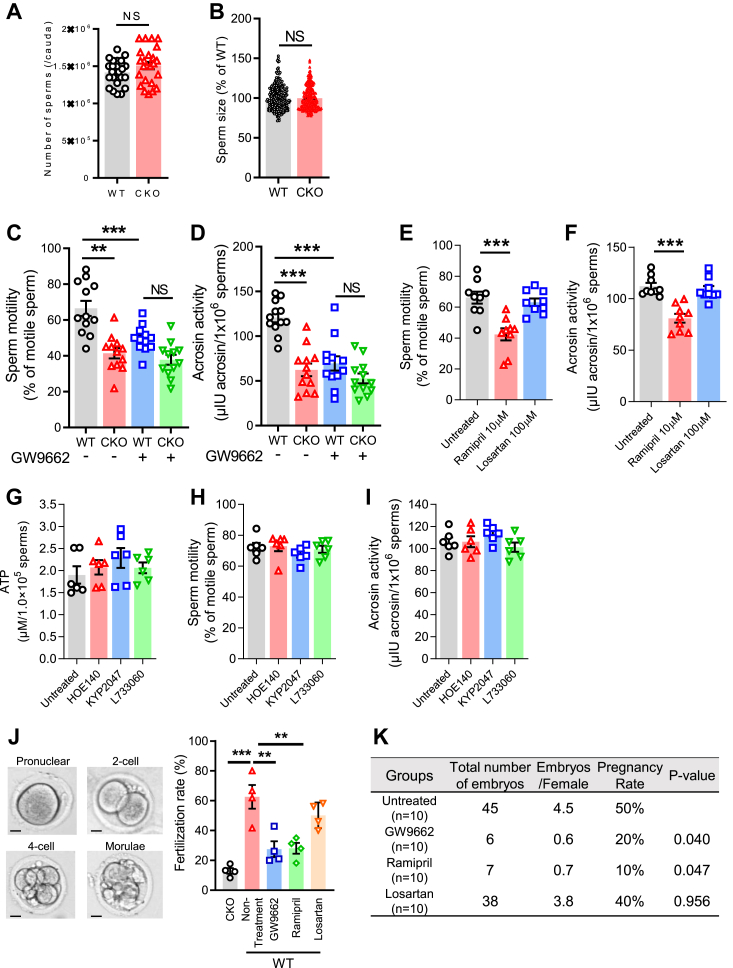
**The role of tACE in sperm physiological functions.***A*, the total number of sperm per cauda in WT and CKO mice. *B*, measurement of sperm length using image-J software. Each *dot* represents a sperm. *C*, measurement of sperm motility. Sperm motility video was shown in Video S1. *D*, measurement of sperm acrosin activity. *C* and *D*, groups of samples were treated for 12 h with 10 mM GW9662 as indicated (n = 12/group). *E* and *F*, measurement of sperm motility (*E*), and acrosin activity (*F*) ± treated for 12 h with either 10 μM ramipril or 100 μM losartan (n = 9/group). *G*–*I*, measurement of ATP production, motility, and acrosin activity. Groups of WT mice were treated with the drugs i.p. (1 dose/day) for 5 days before sperm isolation as follows: bradykinin 2 receptor (B2R) antagonist HOE-140 at 100 μg/kg/day, neurokinin 1 (NK1) receptor antagonist L-733060 at 20 mg/kg/day, and POP inhibitor KYP-2047 at 10 mg/kg/day (n = 6/group). The drugs were continued during the experiment (10 μM HOE-140, 10 μM L-733060 and 50 μM KYP-2047). *J*, *In vitro* fertilization rate of CKO and WT sperm. *Left*: representative image of embryos at different stages (Pronuclear, 2-cell, 4-cell, and Morulae). The scale bar represents 10 μm. *Right*: the fertilization rate was calculated as the number of two cell embryos divided by the number of total oocytes. *K*, measurement of *in vivo* fertilization rate. Table shows pregnancy rates, embryos per female mice, and total number of embryos at E14.0 after artificial insemination. *G* and *H*, groups of mice were treated with 40 mg/l ramipril or 600 mg/l losartan in drinking water for one week before sperm isolation. For PPARγ blockade, isolated sperm were incubated with 10 mM GW9662 for 12 h *A*–*H*, data are presented as means ± SEM. A one-way ANOVA with Bonferroni’s correction for multiple comparisons was used to analyze group comparisons, and data are presented as means ± SEM. NS, no significance. ∗∗*p* < 0.01 and ∗∗∗*p* < 0.001. CKO, C-domain KO; POP, prolyl oligopeptidase; PPARγ, peroxisome proliferators–activated receptor gamma; tACE, testis angiotensin-converting enzyme.

Since the tACE-PPARγ axis controls energy metabolism in sperm, we evaluated whether PPARγ mediates the effect of tACE on sperm physiological function. First, we determined the effect of tACE on the number of sperm. The number of sperm in CKO mice was roughly the same as in WT mice (∼1.5 × 10^6^/cauda, Figure 6*A*). The sperm size was also similar in CKO and WT mice (Fig. 6*B*). Next, we measured sperm motility since ATP plays a crucial role in it (31). The sperm motility of CKO sperm is significantly lower than that of WT sperm, but this difference was not observed in the presence of the PPARγ inhibitor GW9662 (Fig. 6*C* and Video S1). Since acrosin, a sperm-specific acrosomal proteinase, has an essential role in the fertilization process that is dependent on mitochondrial energy (32), we assessed whether tACE affects acrosin activity. We found a significant difference in acrosin activity, which averaged 40% lower in CKO sperm than the WT sperm (62.3 ± 6.7 mIU/1.0 × 10^6^ sperm (CKO) *versus* 102.9 ± 5.1 mIU/1.0 × 10^6^ sperm (WT), *p* < 0.01) (Fig. 6*D*). Again, no difference was observed between CKO and WT sperm with PPARγ blockade (Fig. 6*D*). To verify the role of tACE and Ang II, we performed these assays with either WT sperm treated with or without ramipril (an ACE inhibitor) or losartan (an Ang II AT1 receptor antagonist) for 12 h. While ramipril treatment significantly reduced sperm motility and acrosine activity, no effect was found with losartan treatment (Fig. 6, *E* and *F*). Further, blocking other known ACE-mediated peptide pathways, such as bradykinin/bradykinin 2 receptor (B2R), substance p/NK1R, and Ac-SDKP, had no effects on ATP production, motility, and acrosin activity of WT sperm (Fig. 6, *G*–*I*).

To determine the effect of tACE and PPARγ on fertilization, the rate of CKO and WT sperm fertilization was determined using *in vitro* fertilization (IVF). To block PPARγ, sperm were pretreated for 12 h with or without GW9662. Sperm were then transferred into a culture drop containing cumulus-oocyte complexes collected from the oviducts of WT mice. At 6 h after IVF, the rate of fertilization was measured by comparing the number of zygotes to the total number of eggs. Zygotes developed into 2-cell, 4-cell, and morulae stage embryos at 24 h after the IVF as shown in the images (Fig. 6*J*). The CKO sperm showed an average fertilization rate of 12.9 ± 1.65% as compared to 62.5 ± 6.9% for WT sperm. We found that treatment with GW9662 significantly reduced the rate of fertilization of WT sperm (equivalent to CKO) (Fig. 6*J*). Similar experiments were conducted with ramipril or losartan treatment. As with CKO sperm or PPARγ blockade, ramipril treatment also reduced the rate of fertilization of WT sperm (Fig. 6*J*). In contrast, there was no effect of losartan treatment on sperm fertilization.

To determine *in vivo* fertilization, we performed an artificial insemination (AI) test. To block tACE and PPARγ, WT male mice were administered ramipril or GW9662, and sperm were prepared in the presence of these drugs. AI was performed with WT female mice. Normal WT sperm produced a 50% pregnancy rate, but tACE or PPARγ inhibition reduced it to less than half (10–20%) (Fig. 6*K*). We also noted a significant reduction in the number of embryos/pregnancies with ramipril or GW9662 treatment, while no effect of losartan was observed.

### The effect of tACE inhibition on the metabolism and function of human sperm

**Figure 7:**
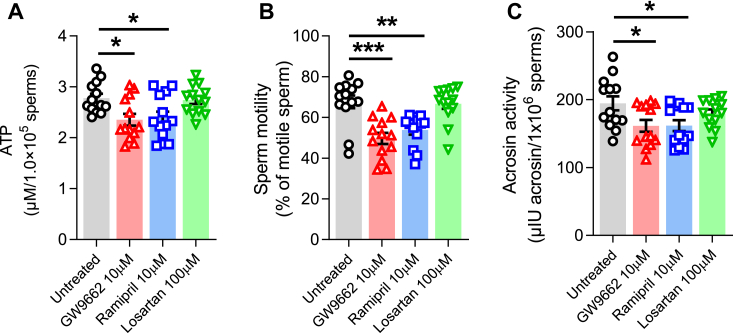
**The metabolic and physiologic effect tACE in human sperm.** Human sperm were treated for 12 h with either 10 μM GW9662 or 10 μM ramipril or 100 μM losartan and then (*A*) production of ATP, (*B*) motility, and (*C*) acrosine activity were determined as described in the Experimental procedures. Untreated samples were used as a control. Sperm representative motility video is shown in Video S2. Data are presented as means ± SEM (n = 13/group). An one-way ANOVA with Bonferroni’s correction for multiple comparisons was used to analyze group comparisons. ∗*p* < 0.05, ∗∗*p* < 0.01, and ∗∗∗*p* < 0.001. tACE, testis angiotensin-converting enzyme.

Finally, a pilot clinical study was conducted to examine the role of tACE in human sperm and validate our findings in mice. Sperm collected from 13 healthy volunteers at our fertility clinic were washed, counted, and prepared for the analysis as described in Experimental procedures. Sperm were treated for 12 h with either GW9662 or ramipril or losartan. We found that inhibition of tACE or PPARγ significantly suppressed ATP production in human sperm (Fig. 7*A*). Consistent with low ATP, GW9662 or ramipril treatment also decreased biological functions of human sperm with lower motility, and acrosin activity as compared to the untreated group (Fig. 7, *B* and *C*, Video S2). However, AT1R blockade by losartan had no effect on human sperm. These findings suggest that even short-term treatment with ACEi may reduce sperm's ability to generate ATP and their physiological functions, and that long-term effects of these drugs should be evaluated in patients as they may affect male fertility.

## Discussion

Sperm motility and function are governed by cellular ATP levels, and mitochondrial dysfunction suppresses sperm motility (33). There are two major metabolic pathways, glycolysis and oxidative phosphorylation, to generate ATP in sperm. Oxidative phosphorylation occurs in the midpiece, while glycolysis occurs in the head and flagellum of sperm (34). Indeed, the head and flagellum lack respiratory enzymes and ATP is only produced by glycolysis (11, 35), while in midpiece, ATP is mainly produced by oxidative phosphorylation. Mitochondrial respiration is positively correlated with ATP production and sperm motility/velocity (11, 35).

This study demonstrates that tACE affects sperm metabolism. What is striking is that tACE is required for maintaining metabolic intermediates. A critical function is the regulation of cellular ATP. Based on mass spectrometry and chemical analysis, this is linked to the catalytic activity of tACE since its catalytic inactivation (CKO) reduced ATP levels. Further, treatment of mice with an ACEi reduced sperm ATP. In contrast, an Ang II AT1 receptor antagonist does not affect sperm ATP. It has been established that ACE is a promiscuous peptidase that has hundreds of substrates (36), but the effect of ACE on sperm is not due to angiotensin peptides, given the repeated ineffectiveness of an AT1 receptor antagonist and genetic ablation of all angiotensin peptides (angiotensinogen KO) on sperm motility and fertilization (2). In contrast, some studies indicate that the presence of Ang II in semen plasma stimulates sperm capacitation, and AT1 receptor blockade reduces sperm motility in mice (37, 38, 39, 40, 41).

Given that ACE can affect sperm ATP, we examined metabolic pathways that may be responsible. Sperm use both glycolysis and oxidative phosphorylation for their energy needs. The mitochondria form a compact helix of 50 to 75 pieces of wrapped mitochondrial sheath in the sperm midpiece, which is essential for fertility in both humans and mice (42, 43). It has been shown that ATP_OxPhos_ is involved in sperm maturation, motility (activation), and fertilization, while ATP_Glyco_, in the head and flagellum, is involved in capacitation and motility (hyperactivation) (34, 44). ATP_OxPhos_ induced activation of motility is required for hyperactivation of motility by ATP_Glyco_ (13, 34). In sperm, tACE is expressed in both the head (the acrosomal region, the equatorial segment, and the postacrosomal region) and midpiece (45, 46). Our analysis revealed that CKO sperm exhibited a downregulation of metabolic intermediates of the Krebs cycle, particularly citric acid, *cis*-aconitic acid, NAD, α-ketoglutaric acid, succinate, and L-malic acid. It appears that tACE increases sperm ATP by activating oxidative metabolism, rather than glycolysis as determined by oxygen consumption analysis of CKO *versus* WT sperm. Further, our analysis revealed that in sperm with tACE inactivation, there is downregulation of some mitochondrial enzymes critical for the Krebs cycle and electron transport chain, including ODO1, CISY, MDHM, HXK1, SDHA, FUMH, CPT2, QCR2, and ATPA. Predictively, these data are consistent with observations that the maximal rate of oxidative respiration is significantly decreased in CKO sperm. Thus, mitochondrial energy production is directly correlated with ACE activity.

Further, we analyzed how tACE induces the level of mitochondrial proteins and ATP. PPARγ transcription factor regulates energy homeostasis in sperm (24, 25, 26). It is also well-known that PPARγ plays a critical role in sperm motility, and fertilization (47). Generally, PPARγ interacts with a coactivator and stimulates transcription of several biological response related genes that play a role in adaptive thermogenesis, mitochondrial biogenesis, and oxidative metabolism (48). Interestingly, PPARγ is also mainly localized in the sperm midpiece and postacrosomal region and is not expressed in the sperm head and flagellum (17). It is important to note that only PPARγ from the PPAR family is significantly decreased in asthenospermia patients whose sperm show low motility (23, 24). Our findings suggest that the lack of tACE causes critically low levels of PPARγ in CKO sperm. To understand the mechanism of how tACE maintains high PPARγ levels, there could be two possibilities: either tACE increases PPARγ transcription or it increases translation including protein stability. Our data suggested that tACE influences PPARγ transcription with significantly low PPARγ mRNA in CKO sperm than the WT sperm. Generally, sperm is considered transcriptionally inert because it gets repressed due to sperm chromatin packaging during spermiogenesis. However, the various mRNAs needed during the stages of spermiogenesis are transcribed in advance before nuclear transcription is terminated (28). Therefore, the regulation of PPARγ through tACE could be at the early stages of sperm development such as spermatids or spermatocytes. Several reports demonstrated that transcription is terminated gradually with the compaction of chromosomal structure. In fact, recent studies revealed that the PPARγ mRNA level is positively correlated to sperm motility (24). These reports support our findings that tACE may regulate PPARγ at the mRNA level.

Concerning PPARγ protein stability, ribosomal complexes are not sufficiently present to support mRNA translation in sperm, but some mRNAs can be translated to support sperm function (49, 50). Sperm also has an ubiquitin–proteasome system for capacitation (51). To assess the role of tACE on PPARγ stability, we used cycloheximide to inhibit translation. PPARγ stability was not different between WT and CKO sperm. Also, the proteasomal degradation rate is similar between WT and CKO sperm as measured using proteosome inhibitor MG-132. Thus, these observations suggest that tACE regulates PPARγ at the RNA level before translation. However, further studies are required to identify at which stage of sperm development tACE acts and how it affects PPARγ mRNA levels.

By using PPARγ antagonist or agonist, we have demonstrated that PPARγ mediates the effect of tACE on sperm metabolism. While PPARγ blockade eliminates the difference between CKO and WT sperm in terms of ATP and mitochondrial protein level, agonist treatment significantly increased ATP production in WT sperm. Agonist had no significant effect on CKO sperm, perhaps due to a very low PPARγ level. Since PPARγ acts as an E3 ubiquitin ligase as well as a transcription factor (52), further study is required to verify the mechanism how PPARγ regulates mitochondrial protein.

Sperm is a special cellular system. There is no cell line representing sperm nor an established protocol for genetic manipulation of these cells. To confirm the role of PPARγ in tACE-mediated energy production, we have tried extensively to transfect sperm with PPARγ plasmid or anti-PPARγ siRNA, but it seems impossible to transfect these cells. Due to this limitation, we cannot further validate our findings through genetic manipulation of PPARγ. However, other studies using epigenetic/genetic approaches have shown that PPARγ-mediated energy metabolism is critical for sperm physiological function and male fertility in both mice and humans (23, 24, 25, 26, 27). Therefore, we can expect any reduction of PPARγ by tACE in sperm will reduce sperm metabolic activity and functions. Similarly, in other cellular systems, such as HEK-293 cells, which can be genetically manipulated, ACE overexpression under a cumate-inducible promoter strongly induces ATP (53). In contrast, ACE overexpression had no effect on ATP production in these cells with PPARγ silencing (Fig. S5). We posit that ACE activates a generalized mechanism—OXPHOS—energy production across different cellular systems (*e.g.*, sperm or epithelial cells) through increasing PPARγ level. However, genetic approaches need to be established for further validating a direct involvement of PPARγ in tACE-mediated energy production in sperm.

Ang II is an important product of the ACE C-domain, which is well-known for regulating blood pressure. However, male mice lacking angiotensinogen have normal fertility (2), indicating that Ang II or any Ang peptides do not mediate tACE function in sperm fertilization. In agreement with this observation, inhibition of tACE, but not the Ang II AT1 receptor, reduced sperm ATP and biological function. These results are similar to our previous findings that Ang II AT1 receptor does not meditates the metabolic and immune functions of ACE in myeloid cells (18, 20). We also found that blocking three other well-studied ACE-mediated peptide pathways (bradykinin/B2R, substance p/NK1R, and Ac-SDKP) showed no effects on sperm function. At present, we do not know the peptides that regulate PPARγ and sperm metabolism. Identifying this peptide is required to reveal the precise mechanism of how tACE regulates male fertility.

ACEi are used by millions of patients for the treatment of hypertension or cardiovascular diseases, and some of them are young reproductively active males. Moreover, men have a higher prevalence of hypertension than women among adults aged 18 to 39 (9.2% of the total population compared with 5.6%, respectively) and 40 to 59 (37.2% compared with 29.4%, respectively) (10). Therefore, it is clinically important to examine the effect of these drugs on male fertility. Our study examines the short-term effects of ACEi or ARB on sperm fertilization as measured by IVF and artificial insemination. Treatment with ACEi resulted in a significant reduction in pregnancy in mice. In humans, as in mice, *in vitro* treatment with ACEi reduced ATP levels, motility, and acrosin activity in sperm, while ARB treatment had no effect on sperm physiology. In fact, a clinical study in patients enrolled in an IVF program reported that sperm with reduced tACE level failed to fertilize ova (54). Although ACE affects many physiological systems, any reduction in sperm metabolism and fertilization might be expected to contribute to an increased risk of male infertility. Therefore, our findings have clinical implications and suggest that the long-term effect of ACEi on male fertility should be further explored.

## Experimental procedures

### Mouse and human sperm collection

All animal and human studies were approved by the Institutional Animal Care and Use Committee or Institutional Research Ethics Board of Cesar-Sinai Medical Center. The Declaration of Helsinki principles were followed in all human studies.

CKO and WT control mice were used in this study. CKO mice were generated by using point mutation to inactivate the catalytic activity of tACE or the C-domain of sACE, as described previously (1, 36, 55). Male C57BL/6J mice (8 weeks old) with proven fertility were used for sperm collection and the sperm were collected by placing cauda epididymidis in prewarmed Enhance W media for 1 h and allowing sperm to swim out, as described previously (56, 57). After filtering through a 100 μm filter, sperm suspension was layered on a two-layer (40–80%) gradient (PureSperm 40/80, Nidacon) in a 14 ml tube. The tubes were centrifuged at 1000*g* for 20 min at room temperature. After sperms were collected from the supernatant and were resuspended in Enhance W media, the sperm suspension was counted using a hemacytometer.

Human sperm were donated by thirteen healthy male participants at Cedars Sinai fertility clinic. Ejaculated samples were washed twice with Enhance W media and then filtered through a 100 μm filter to remove substrates present in the epididymal fluid. A hemocytometer was used to count sperm after centrifuging samples and dissolving pellets in fresh media. The number of sperm isolated from everyone ranged from 15 to 100 million. A large-bore pipette tip was used in all procedures to prevent damage to sperm membranes.

### Metabolite array by mass spectrometry

After collection and washing, sperm pellets containing ten million purified sperm were treated with 700 ml of cold 40% acetonitrile, 40% methanol, and 20% water. Then samples were vortexed vigorously for 5 min at 4 °C and spun at 10,000*g* for 10 min at 4 °C. The supernatant was removed and placed in a SpeedVac until dry. To resuspend, 20 ml of methanol was added, followed by vortexing, and finally, 80 ml of water was added along with a final vigorous vortex. For LC–MS analysis, 4 ml of sample volume was injected. Analysis method was described previously (20). Briefly, cell metabolite extractions were analyzed with an Agilent 6470A triple quadrupole mass spectrometer, operating in negative mode, connected to an Agilent 1290 ultra-high-performance liquid chromatography (UHPLC) system (Agilent Technologies). The analytical column used was an Agilent ZORBAX RRHD Extend-C18 1.8 mm 2.1 × 150 mm coupled with a ZORBAX Extend Fast Guard column for ultra-high-performance liquid chromatography Extend-C18, 2.1 mm, 1.8 mm. The MassHunter Metabolomics dMRM database and method was used to scan for polar metabolites within each sample (Agilent Technologies). The resulting chromatograms were visualized in Agilent MassHunter Quantitative Analysis for QQQ (Agilent Technologies). The final peak areas were manually checked for consistent and proper integration.

### MitoPlex assay

Mitochondrial proteomic analysis was described previously (20, 21). Briefly, sperm pellets (n = 5 per mouse genotype, WT *versus* CKO) were lysed in 8 M urea dissolved in 50 mM Tris–HCl buffer, pH 8.0. Lysis was facilitated by high-pressure treatment on a Pressure BioSciences barocycler (model 2320EXT), with 60 1-min cycles consisting of 50 s at 45,000 p.s.i. followed by 10 s at atmospheric pressure. Peptides were prepared as previously described (20). A total of 8 mg of digested peptides, injected twice as duplicate technical replicates, were separated on a Prominence UFLCXR HPLC system (Shimadzu Corp) with a Waters Xbridge BEH30 C18 2.1 mm × 100 mm, 3.5-mm column flowing at 0.25 ml/min and 36 °C coupled to a QTRAP 6500 (SCIEX). Raw data were processed using the Skyline software package (Skyline Daily, version 19.1.1.309) to select peak boundaries and quantify the area under the curve for each fragment monitored.

### Ingenuity pathway analysis

Significantly different metabolites and mitochondrial proteins (*p* < 0.01 and *p* < 0.05, respectively) between CKO and WT sperm were imported to the Ingenuity Pathway Analysis Tool (IPA Tool; Ingenuity Systems, Redwood City, CA, USA; http://www.ingenuity.com), and then mapped to well-known biological networks using the Ingenuity Pathway Knowledge Base derived from known functions and interactions of genes published in the literature.

### Functional and pathway enrichment analysis of mitochondrial proteins

MitoPlex proteins were analyzed for their functional enrichment and biological processes using Gene Ontology and Kyoto Encyclopedia of Genes and Genomes pathway analysis, which used DAVID database (https://david.ncifcrf.gov/), an online tool for gene annotation, function visualization, and large volume data integration (58, 59). To describe gene product attributes, Gene Ontology clusters included three complementary biological concepts (biological process, molecular function, and cellular component).

### Western blot analysis

Sperm were lysed with radioimmunoprecipitation assay buffer containing protease and phosphatase inhibitors (Thermo Fisher Scientific). The polyvinylidene difluoride membranes were incubated with specific antibodies including CISY rabbit mAb (Cell Signaling Technology, 14309S), FUMH rabbit polyclonal antibody (Proteintech, 11375-1-AP), ODO1 rabbit polyclonal antibody (Proteintech, 15212-1-AP), SDHA rabbit mAb (Cell Signaling Technology, 11998T), CPT2 rabbit mAb (Abcam, ab231162), ATPA mouse mAb (Invitrogen, 459240), GAPDH rabbit polyclonal antibody (Sigma-Aldrich, G9545), PPARγ rabbit polyclonal antibody (Invitrogen, PA3-821A). Protein bands were measured using an Odyssey Infrared Imaging System (ODYSSEY CLx, Li-COR). The fluorescence intensity was evaluated using Image Studio Lite version 5.2 (https://www.licor.com/bio/image-studio-lite/).

### OCR and ECAR assessment

For OCR assay, Agilent MitoXpress Xtra (MX-200-4, Agilent) reagent was reconstituted in 1 ml growth medium then diluted in 10 ml prewarmed growth medium. For ECAR assay, Agilent pH-Xtra reagent (PH-200-4, Agilent) was reconstituted in 1 ml distilled water then diluted in 10 ml prewarmed respiration buffer (PH-200-4, Agilent). Sperm (5 × 10^5^ sperm/well) were centrifuged (600*g* for 5 min) onto 96-well plates coated with Cell-Tak (Corning, catalog no. C354240) according to the manufacturer’s instructions. After 12 h incubation at 37 °C with or without 10 mM GW9662, medium was replaced in each well with 100 μl of the MitoXpress Xtra reagent or pH-Xtra reagent then sealed by overlaying with 100 μl prewarmed HS oil (MX-200-4, Agilent) in OCR assay. The plates were then immediately measured kinetically on a FLUORstar (BMG Labtech) plate reader (prewarmed to 37 °C, Ex 380 nm, Em 650 nm) for 120 min. Oligomycin (2 μM), FCCP (500 nM), antimycin A (1 μM) with rotenone (200 nM) (AA/ROT), glucose (10 mM) and 2-deoxy-d-glucose (2DG, 50 mM) were added acutely to the wells, and the kinetic data were analyzed by performing linear regression over the linear part of the kinetic data. Respiratory parameters and ATP production rates were calculated as described previously (20, 29, 30).

### Quantitative real-time PCR

The mRNA was extracted from sperm using RNeasy Kit (Qiagen). Quantitative real time PCR was performed using OneStep RT-PCR Kit (Qiagen), according to the manufacturer’s instructions. Specific primer for PPARγ (Mm00440940_m1) was purchased from Thermo Fisher Scientific. The mRNA levels were normalized to the internal control β-actin (Mm02619580_g1). Group of mice were treated with 40 mg/l ramipril for 1 week to block tACE activity before isolation of sperm.

### Protein stability assays

To assess PPARγ protein stability, sperm were seeded in 6-well plate (1.0 × 106 sperm/well) and incubated with a translation inhibitor cycloheximide (20 μg/ml) or proteasome inhibitor MG-132 (50 μM). At the indicated time points, sperm were harvested and PPARγ protein levels were analyzed by Western blot.

### Analysis of intracellular ATP content and ATP production rate

Mouse or human sperm were seeded in 96-well plate (5 × 10^5^ sperm/well) and incubated for 12 h with or without GW9662 (10 μM), pioglitazone (10 μM), ramipril (10 μM), and losartan (100 μM). ATP was measured using the Cell Titer-Glo 2.0 kit (Promega) as recommended by the manufacturer’s protocol.

### Measurement of mitochondrial number, size, and morphology

For flow cytometry, sperm were suspended in prewarmed staining solution (0.1% bovine serum albumin in PBS) containing MitoSOX Red (Thermo Fisher Scientific). After incubation for 10 min at 37 °C and 5% CO_2_, sperm were rinsed three times with prewarmed PBS and then analyzed with flow cytometry (CYTEK NL-3000). Data were analyzed with FlowJo version 10.8.1 (https://www.flowjo.com/solutions/flowjo/downloads/previous-versions).

For confocal microscopy, sperm were seeded on 8-well Chamber Slide (Nunc) with Cell-Tak and then centrifuged for 1 min at 700*g*. After incubation with MitoSOX Red for 10 min at 37 °C and 5% CO_2_, sperm were washed with prewarmed PBS and then with PBS containing 4% formaldehyde for 30 min. After rinsing with PBS three times, the sperm were permeabilized with 0.1% Triton X-100 at room temperature for 20 min and washed three times with PBS. They were then incubated with Phalloidin-Green (Hello Bio) for 30 min, rinsed three times with PBS, and incubated with ProLong Gold Antifade Reagent with 4′,6-diamidino-2-phenylindole (Life Technologies) for 2 h. Fluorescence localization was observed using a Leica Stellaris 8-STED Super-resolution Confocal Microscope (Leica). Image analysis for the fluorescence localization was performed using LAS AF Lite (Leica). Data were processed by CellProfiler and analyzed by GraphPad Prism software (https://www.graphpad.com/features).

### Acrosin activity assay

Acrosin activity was assessed by the method of Aquila *et al.* (25). Purified mice or human sperm were washed with Enhance W media and centrifuged at 700*g* for 3 min. Sperm were resuspended (1 × 10^7^ sperm/ml) in different tubes and incubated for 12 h with or without either 10 μM GW9662 or 10 μM ramipril or 100 μM losartan. After incubation, sperm were centrifuged at 700*g* for 10 min. Then sperm were resuspended in 1 ml of substrate–detergent mixture (23 mmol/L Nα-benzoyl-_DL_-arginine *p*-nitroanilide in dimethyl sulfoxide and 0.01% Triton X-100 in 0.055 mol/L NaCl, 0.055 mol/L Hepes at pH 8.0, respectively) and incubated for 3 h at room temperature. After incubation, 100 μl benzamidine (0.5 M) was added to each of the tubes and then centrifuged at 1000*g* for 30 min. The supernatants were collected and the acrosin activity was measured spectrophotometrically at 410 nm. The acrosin activity was determined as described by Aquila *et al.* (60) and presented as μIU/10^6^ sperm.

### Inhibitors

To investigate the role of the ACE substrate pathways bradykinin/B2R, substance p/NK1R and Ac-SDKP on sperm function, WT mice were treated i.p. for 5 days (1 dose/day) before sperm isolation, as follows: the B2R blocker HOE-140 (Sigma-Aldrich) at 100 μg/kg/day, Neurokinin 1 (NK1) receptor blocker L-733060 (Tocris) at 20 mg/kg/day, or prolyl oligopeptidase inhibitor KYP-2047 (Sigma-Aldrich) at 10 mg/kg/day.

### Sperm motility

Using a light microscope, sperm motility was measured using live videography and real-time movement of sperm. Motility of sperm is calculated as a percentage of total motile sperm using a LPIXEL Image J Plugin. Videos of sperm motility are shown in Videos S1 and S2. To verify the role of tACE, Ang II and PPARy, groups of samples were treated for 12 h with either 10 μM ramipril or 100 μM losartan or 10 μM GW9662 before analysis.

### *In vitro* fertilization

IVF was performed by the method of Nakao *et al.* (61). Female mice were intraperitoneally injected 7.5 IU/100 μl pregnant mare serum gonadotropin (PMSG, Prospec-Tany Technogene). At 48 h after PMSG injection, mice were intraperitoneally injected 7.5 IU/100 μl human chorionic gonadotropin (hCG, Prospec-Tany Technogene). At 15 h after the hCG injection, their oviducts were collected and transferred to human tubal fluid medium on Ovoil in 60 mm^3^ dish. Cumulus–oocyte complexes were collected from the oviducts and transferred into a drop of sperm suspension (5 × 10^5^ sperm) and covered with Ovoil. After 6 h incubation at 37 °C in 5% CO_2_, the oocytes were collected and washed three times in 100 μl drops of human tubal fluid covered with Ovoil. After 24 h co-incubation, the fertilization rate was calculated by the formula: fertilization rate (%) = the total number of two-cell embryos/the total number of oocytes. Groups of male mice were treated with 40 mg/l ramipril or 600 mg/l losartan in drinking water for a week before sperm isolation. For PPARγ blockade, isolated sperm were pretreated with GW9662 for 12 h before inoculation with oocytes.

### Artificial insemination (AI)

After superovulation by PMSG (7.5 IU) and hCG (7.5 IU) as described in IVF section, purified sperm were transferred to the female mice (2 × 10^6^ sperm/50 μl Enhanced W) using a blunt 19-gauge needle inserted through the vagina. The transfer was performed without the use of anesthesia or analgesia.

### Cell culture

HEK-293 cells were obtained from the American Type Culture Collection and cultured at 37 °C in Dulbecco’s modified Eagle’s medium supplemented with 10% fetal bovine serum in a humidified atmosphere containing 5% CO2. To create HEK-ACE cell line, HEK-293 cells were stably transfected with PiggyBac plasmid expressing ACE under a cumate-inducible promoter (Fig. S5*A*) using Lipofectamine LTX (15338030, Invitrogen) according to the manufacturer's protocol. Stable transfected cells were selected with 1 μg/ml puromycin (Sigma) in complete Dulbecco’s modified Eagle’s medium.

The PPARγ siRNA (Cat. # AM16708) was purchased from Invitrogen. Cells were transfected using Lipofectamine RNAiMAX (13-778-150, Invitrogen) and Opti-MEM (31-985-062, Gibco) according to manufacturer recommendations.

## Data availability

All data generated or analyzed during this study are included either in this article or in the supplementary information files.

## Supporting information

This article contains supporting information.

## Conflict of interest

The authors declare that there is no conflict of interest regarding the publication of this article.

## Supporting information

Supporting Table S3

Supporting Videos S1 and S2
