# Supplementary material for: Testicular ACE regulates sperm metabolism and fertilization through the transcription factor PPARγ
Source: J Biol Chem. 2023 Nov 20;300(1):105486. doi: 10.1016/j.jbc.2023.105486 (PMC10788540; doi:10.1016/j.jbc.2023.105486)
Supplement: Supporting Videos S1 and S2 [file mmc4.pptx]

## Slide 1
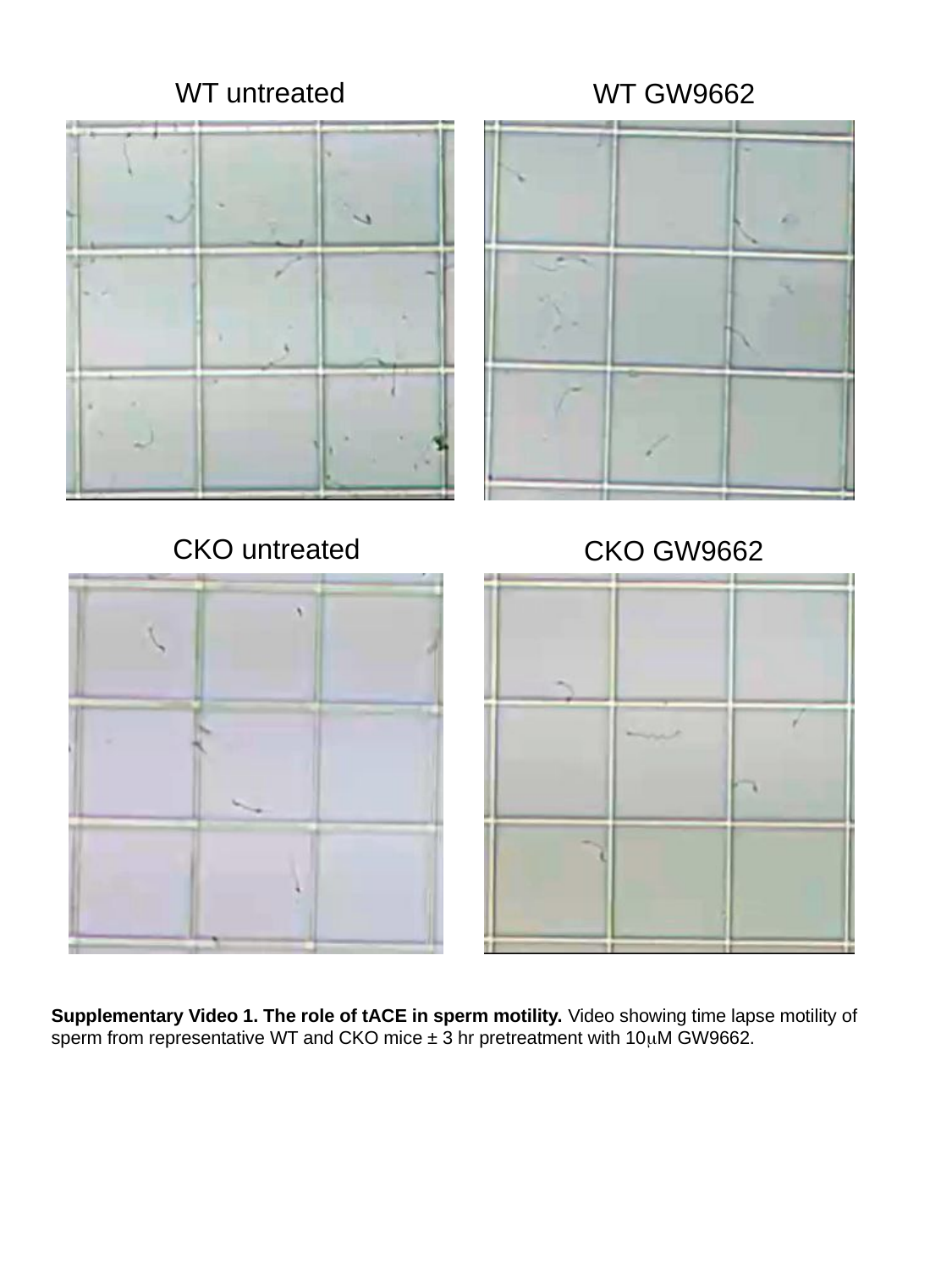

WT untreated
WT GW9662
CKO untreated
CKO GW9662
Supplementary Video 1. The role of tACE in sperm motility. Video showing time lapse motility of sperm from representative WT and CKO mice ± 3 hr pretreatment with 10mM GW9662.

## Slide 2
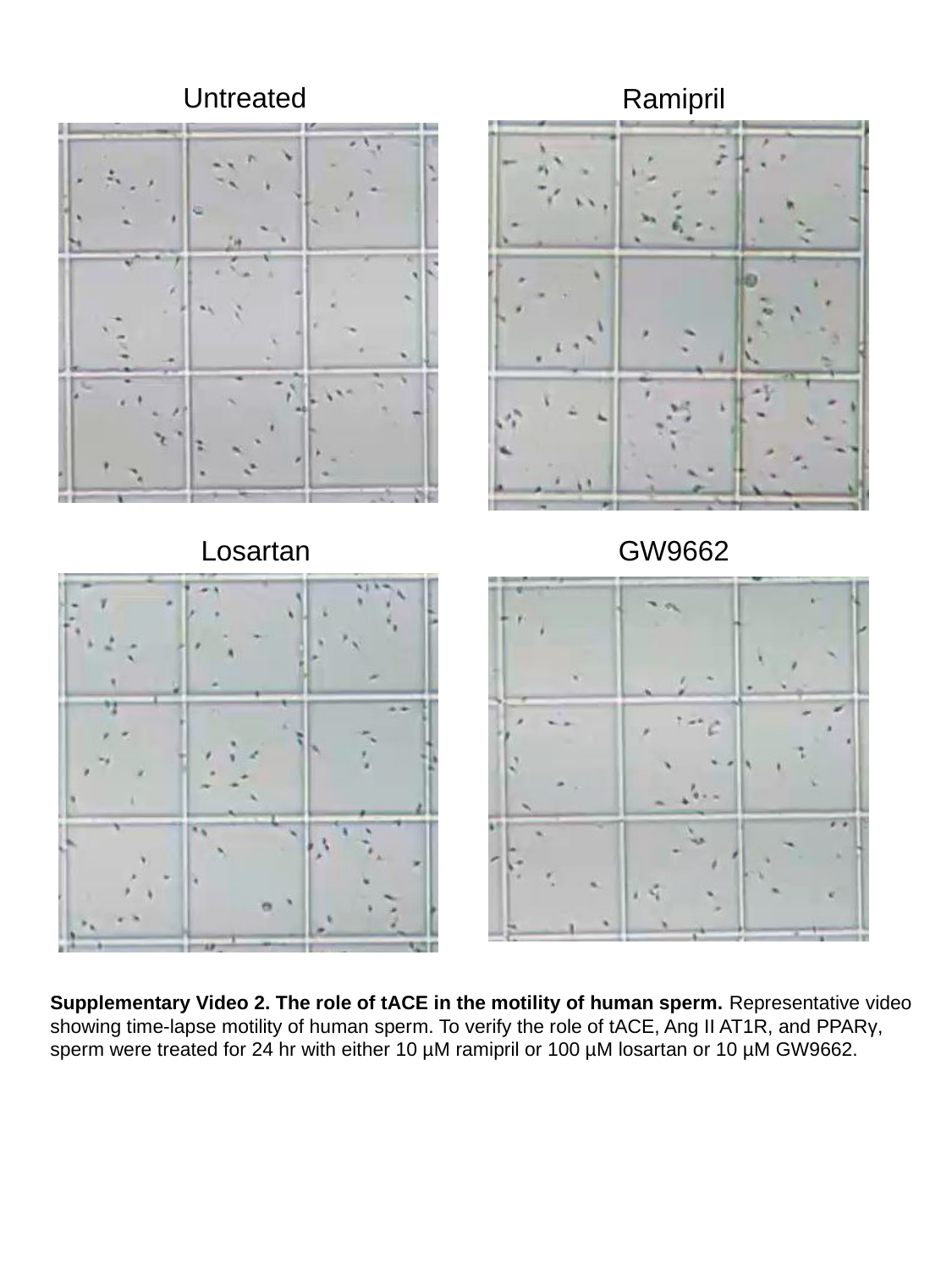

Untreated
Ramipril
Losartan
GW9662
Supplementary Video 2. The role of tACE in the motility of human sperm. Representative video showing time-lapse motility of human sperm. To verify the role of tACE, Ang II AT1R, and PPARγ, sperm were treated for 24 hr with either 10 µM ramipril or 100 µM losartan or 10 µM GW9662.
