# Supplementary material for: Testicular ACE regulates sperm metabolism and fertilization through the transcription factor PPARγ
Source: J Biol Chem. 2023 Nov 20;300(1):105486. doi: 10.1016/j.jbc.2023.105486 (PMC10788540; doi:10.1016/j.jbc.2023.105486)
Supplement: Supporting Figures S1–S5 [file mmc5.docx]

**Supplementary Figure 1. tACE affects mitochondrial protein expression in sperm.** Graphs show mitochondrial proteins that are significantly different between WT and CKO sperm as measured by Mitoplex assay. Data are presented as means ± SEM (n = 5 per group). *p<0.05, **p<0.01, ***p<0.001 determined by two-tail student t-test


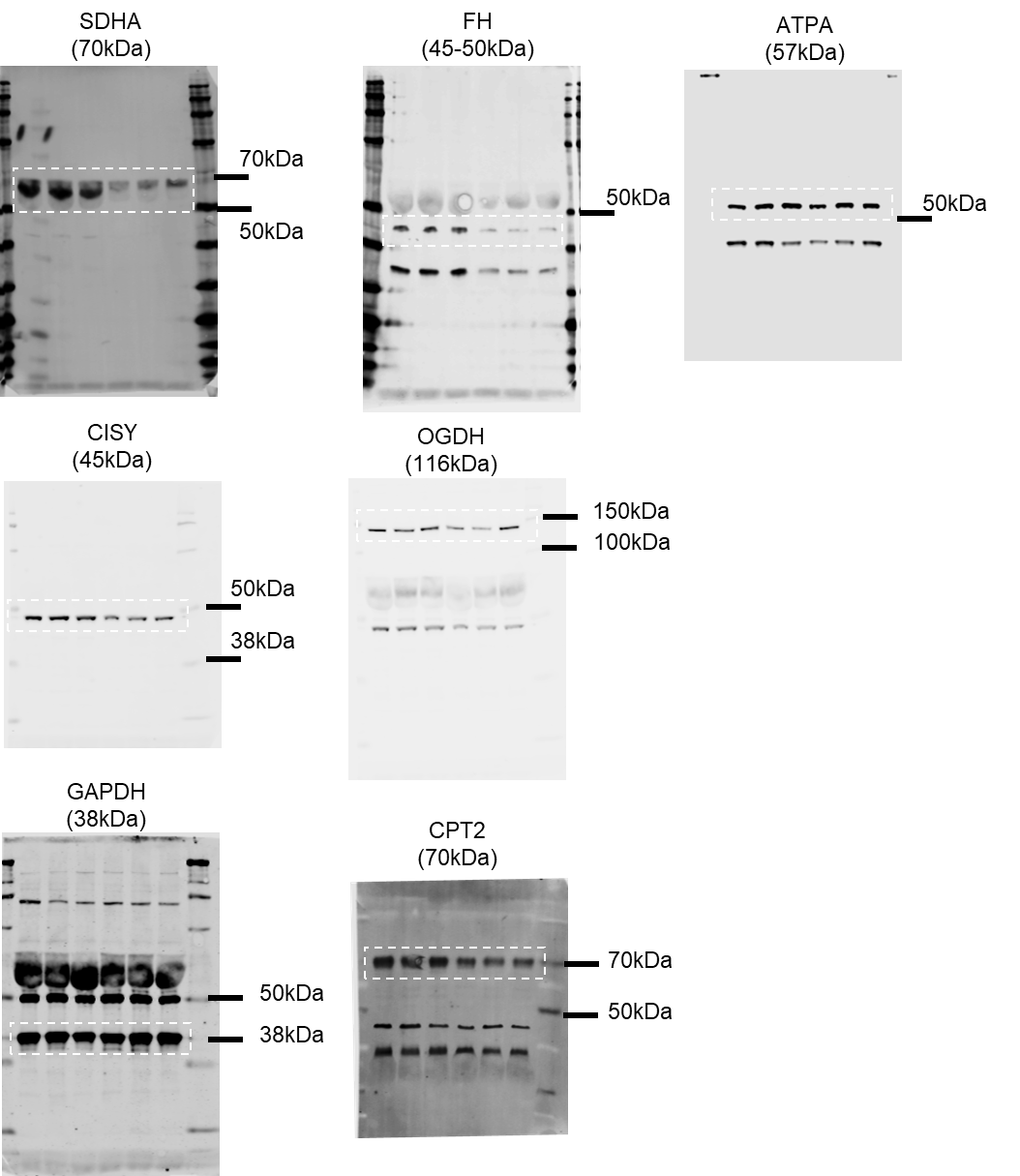


**Supplementary Figure 2. Uncropped Western blot images for Figure 2C.**


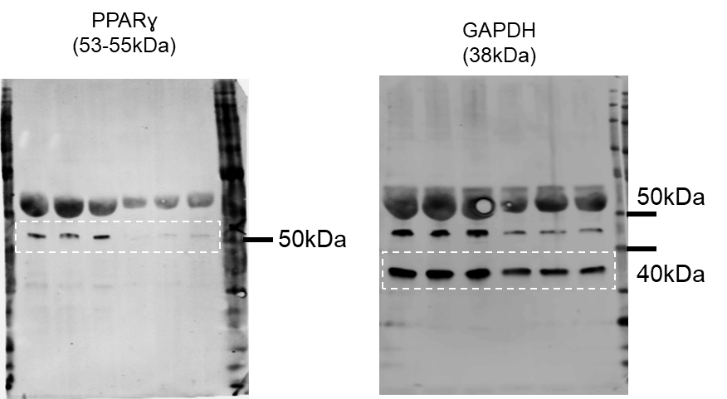


**4A**


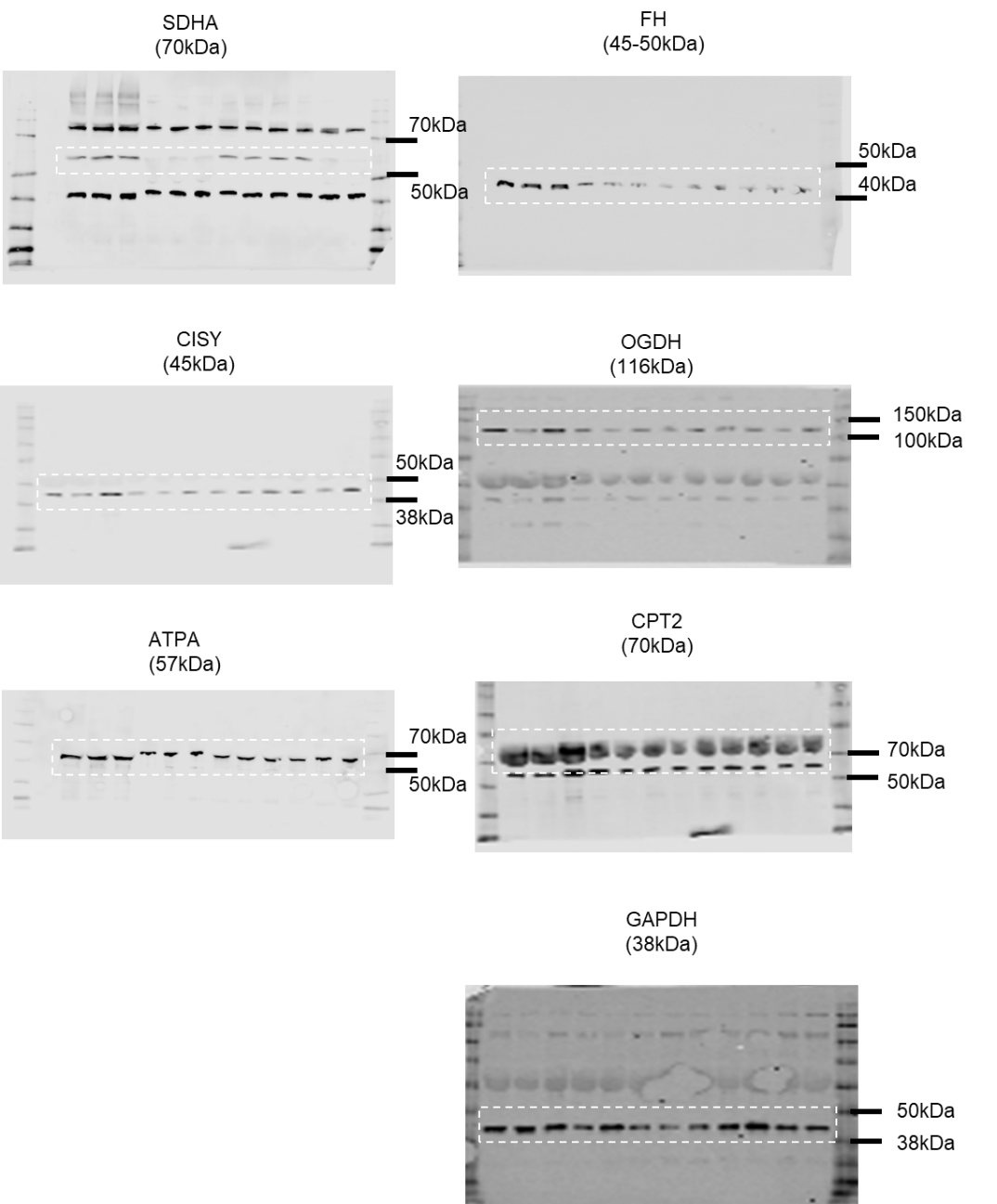
 **4F**

**Supplementary Figure 3. Uncropped Western blot images for Figure 4A** **and** **4F.**

**Supplementary Figure 4. Measurement of sperm PPARg protein stability in CKO and WT mice ± WT mice treated with ramipril (40 mg/L) for one week.** Sperm were cultured and treated with either translational inhibitor cycloheximide (CHX, 20 mg/mL, upper) or proteosome inhibitor MG-132 (50 mM, lower). Sperm were lysed at the indicated timepoints and PPARg protein synthesis or degradation was determined with Western blot analysis. Right panel showing degradation curves for PPARg normalized to PPARg expression levels at 0 hr.

**C**

**B**

**A**

**Supplementary Figure 5.** The analysis of a stable HEK-ACE cell line overexpressing ACE under a cumate-inducible promoter is presented. **(A)** Plasmid construct used for generation of HEK-ACE cell line from HEK-293 cell line. **(B)** Western blot analysis. **(C)** Measurement of ATP by Cell Titer-Glo 2.0 kit (Promega). To induce ACE expression, cells were treated with 1 µM cumate for 48 hr before analysis. For PPARγ silencing, cells were treated with 10 nM anti-PPARγ siRNA along with cumate. Data are presented as means ± SEM. A one-way ANOVA with Bonferroni’s correction for multiple comparisons was used to analyze group comparisons, and data are presented as means ± SEM. ns, no significance. ***P < 0.001.
